# Large Improvement in the Mechanical Properties of Polyurethane Nanocomposites Based on a Highly Concentrated Graphite Nanoplate/Polyol Masterbatch

**DOI:** 10.3390/nano9030389

**Published:** 2019-03-07

**Authors:** Sang-Hyub Lee, Cho-Rong Oh, Dai-Soo Lee

**Affiliations:** Division of Semiconductor and Chemical Engineering, Chonbuk National University, Baekjedaero 567, Jeonju 54896, Korea; shlee87@jbnu.ac.kr (S.-H.L.); ohcho38@naver.com (C.-R.O.)

**Keywords:** graphite nanoplate, polyurethane, nanocomposite, master batch, lyotropic liquid crystalline behavior

## Abstract

In this study, a highly concentrated graphite nanoplate (GNP)/polyol masterbatch was prepared by the exfoliation of natural graphite in an aqueous system using cetyltrimethylammonium bromide and the replacement of aqueous solution with a polyol, viz. poly(tetramethylene ether glycol), and it was subsequently used to prepare polyurethane (PU) nanocomposites by simple dilution. The polyol in the masterbatch efficiently prevented the aggregation of GNPs during the preparation of PU nanocomposite. In addition, the dispersed GNPs in the masterbatch exhibited rheological behavior of lyotropic liquid crystalline materials. In this study, the manufacture and application methods of the GNP/polyol masterbatch were discussed, enabling the facile manufacture of the PU/GNP nanocomposites with excellent mechanical properties. In addition, the manner in which the GNP alignment affected the microphase separation of PU in the nanocomposites was investigated, which determined the improvement in the mechanical properties of the nanocomposites. High-performance PU/GNP nanocomposites are thought to be manufactured from the GNP/polyol masterbatch by the simple dilution to 0.1 wt% GNP in the nanocomposite.

## 1. Introduction

Polymer composites prepared by incorporating inorganic fillers or reinforcements into various organic polymers have been applied for use in automotive parts, sports goods, and electronic devices due to the excellent mechanical properties, dimensional stability, and the functional properties imparted by the fillers. In the last 20 years, polymeric nanocomposites where the dimensions of fillers are of the order of a nanometer have attracted the attention of material scientists, as the nanocomposites showed superior properties to the conventional composites at low concentration of nanofillers. Nanofillers such as clay, carbon nanotube, graphene, and nanocellulose have been studied for the nanocomposites of different polymers for various applications [1,2,3,4,5,6,7,8,9,10,11,12].

Polyurethane (PU) is one of the most versatile polymers and is being developed as a nanocomposite with various nanofillers because it can be applied in various fields such as to foams, coatings, adhesives, and elastomers [13,14,15,16,17,18,19,20,21]. Among various nanofillers for PU nanocomposites, graphene with excellent mechanical, electrical, and thermal properties is considered to be the most promising. Specially, its role in the enhancement of mechanical properties is considered to be the most essential aspect for structural applications. Several studies have reported the enhancement of mechanical properties of the PU nanocomposites by the addition of graphene [19,20,21,22,23,24,25,26,27,28,29,30,31,32,33,34,35]. Typically, graphene oxide (GO) has been widely used for the reinforcement of PU nanocomposites [22,24,26,27,28,29]. However, oxidation inevitably leads to the introduction of considerable defects in the structure and destruction of the *sp*^2^-hybrized carbon atom structure, leading to the decline in the intrinsic properties of graphene [36,37,38,39,40]. In addition, several studies have examined the modification of a graphene surface with organic or polymeric materials to improve its compatibility with PU [23,25,30,31,32,33,34]. Nevertheless, typical chemical oxidation or significant structural destruction must be conducted for such modification. The structural damage to graphene caused by the modification does not completely improve its compatibility with the PU matrix, adversely affecting the large performance enhancement of the PU nanocomposites. Therefore, to prepare high-performance urethane nanocomposite, it is very important to produce graphene with minimum defects and ensure compatibility with PU matrices.

Thermoplastic elastomers (TPEs) have the advantages of thermoplastics and elastomers. Thus various TPEs have been developed to replace vulcanized crosslinked rubbers. Thermoplastic PU elastomer is one of representative TPEs for automotive parts, sports goods, and medical devices such as catheters. The PU elastomers are generally prepared from polyols, diisocyanates, and chain extenders by step-wise addition polymerizations. In general, polyols constitute soft segments while diisocyanates and chain extenders constitute hard segments in the PU after the polymerizations. Due to the limited miscibility of the soft and hard segments of PUs, microphase separation of segments occurs in PUs and the hard segment domains play the role of physical crosslinking [19]. The microphase separations of PUs are affected by the structures and molecular weights of the constituents. In nanocomposites of PUs, nanofillers affect the microphase separation and the mechanical properties of the nanocomposites. Thus, effects of graphenes on the microphase separation of graphene/PU nanocomposites were studied and reported already in the literature [19,20]. Graphenes generally exhibit lyotropic liquid crystalline (LLC) behavior in dispersion due to their discotic natures [41,42,43,44]. Thus, LLC properties of graphenes in the nanocomposites should be investigated to understand the structure–property relationships of the nanocomposites. However, to the best our knowledge, there is no systematic study on the effect of graphene alignment on the microphase separation of PU nanocomposites. 

In this study, for the preparation of PU nanocomposites with high quality graphite, a highly concentrated graphite nanoplate (GNP)/polyol masterbatch was prepared by the exfoliation of natural graphite (NG) in an aqueous system using cetyltrimethylammonium bromide (CTAB), and the replacement of aqueous CTAB solution with a polyol, viz. poly(tetramethylene ether glycol), which was subsequently used to prepare PU nanocomposites. By the simple dilution of the masterbatch, PU/GNP nanocomposites with various GNP concentrations were easily prepared. Notably, the dispersion of GNPs in the polyol exhibited rheological behavior of lyotropic liquid crystalline (LLC) materials due to the discotic nature and considerably affected the microphase separation of PU molecules in the nanocomposites, thereby considerably affecting the mechanical properties of the nanocomposites. 

## 2. Experimental 

### 2.1. Materials

NG (mesh: 50) was purchased from Hyundai Coma (Yecheon-eup, Korea). Potassium hydroxide (80%) was obtained from Dae-Jung (Si-heung, Korea). CTAB, poly(tetramethylene ether glycol) (polyol, MW: 1000 g/mol), 4,4′-diphenylmethane diisocyanate (MDI), and 1,4-butandiol (BD) were purchased from Sigma-Aldrich (Young-in, Korea). All chemicals were used as received.

### 2.2. Preparation of Graphite Nanoplates (GNPs) 

GNPs were prepared by the sonication of NG with CTAB in deionized (DI) water. First, 2 g of NG was mixed with 2000 mL of DI water and 0.25 g of CTAB. Second, this mixture was subjected to ultrasonication for 24 h (power: 300 W). After sonication, the graphite dispersion was subjected to centrifugation at 5000 rpm for 20 min, leading to a CTAB–GNP complex with a few-layer thickness (2–3 nm). Finally, 0.35 mg/mL of GNPs obtained in the supernatant was used for the preparation of the GNP/polyol masterbatch via the replacement of an aqueous solution of CTAB with a polyol, viz. poly(tetramethylene ether glycol). As a control, graphite oxide (GO) was prepared according to the Hummers method [24].

### 2.3. Preparation of GNP/Polyol Masterbatch

To prepare the masterbatch, the GNP solution (0.35 mg/mL) obtained after centrifugation was thoroughly mixed with polyol and an excess amount of aqueous KOH solution at room temperature for 1 h. In this step, the coagulation of CTAB with GNPs and polyol occurred. Subsequently, washing with DI water was repeated at room temperature to remove the residual KOH and CTAB until neutral pH was achieved. The GNP/polyol supernatant was collected after washing and was dried in a vacuum oven at 80 °C for several days to thoroughly remove the residual water, finally yielding the GNP/polyol masterbatch.

### 2.4. Polymerization of Polyurethane (PU)/GNP Nanocomposites

All PU/GNP nanocomposite samples were subjected to polymerization from the prepared GNP/polyol masterbatch. The prepared GNP/masterbatch containing 6 wt% of GNP was diluted by the addition of polyol and directly used for the preparation of the GNP/PU nanocomposites. The concentration of GNPs in the PU nanocomposites was varied from 0 to 0.5 wt%. Hereafter, the samples were referred to as PU-0, PU-0.05, PU-0.1, PU-0.2, and PU-0.5 according to the GNP concentration. To prepare the isocyanate-terminated PU prepolymer, monomeric MDI was melted in a four-neck flask with N_2_ gas purging. Subsequently, the diluted masterbatch with additional polyol was added into the flask, and the mixture was stirred at 60 °C until the isocyanate contents (NCO%) reached the theoretically calculated value (ASTM D1638-74). Then, the amount of the chain extender BD was calculated from the NCO% of the PU prepolymer. For the thermal curing of nanocomposites, a mixture of the PU prepolymer and BD was poured into a glass mold and placed in an oven at 110 °C for 24 h. Table 1 summarizes the sample codes and compositions of the GNP/PU nanocomposites prepared with various GNP concentrations.

### 2.5. Characterization

The concentration of GNPs that were obtained by the exfoliation of NG using CTAB in the water solution was estimated by ultraviolet–visible spectrometry (UV-PC, V-670, JASCO, Easton, MD, USA). The characteristics of GNPs, including surface morphology and thickness, were examined by high-resolution transmission electron microscopy (HR-TEM, JEM-2010, JEOL, Akishima, Japan), Raman spectroscopy (RAMAN, Nanofinder 30, Tokyo Instruments, Tokyo, Japan), and atomic force microscopy (AFM, Nanoscope III, Veeco Instruments, Plainview, NY, USA) in the tapping mode. The thermal properties of GNPs and nanocomposites were examined by differential scanning calorimetry (DSC, Q20, TA Instruments, New Castle, DE, USA) and thermogravimetric analyzer (TGA, Q600, TA Instruments) at heating rates of 10 and 20 °C/min, respectively, under N_2_ environments. Tensile tests were carried out with a universal testing machine (UTM, LR5K Plus, LLOYD Instruments, Bognor Regis, UK) at a pulling rate of 500 mm/min at room temperature. The tensile test was performed at least five times per sample. The degree of hydrogen bonding in the PU nanocomposites was analyzed by Fourier-transform infrared spectroscopy (FTIR, FT/IR-300E, JASCO). Field-emission scanning electron microscopy (FE-SEM, SUPRA 40VP, Carl Zeiss, Oberkochen, Germany) with energy-dispersive X-ray analysis (EDX) was employed for the characterization of the nanocomposite morphologies. The rheological properties of the GNP dispersion polyol and the GNP/PU nanocomposites were characterized using a cone-plate rheometer (Rheometer, AR2000, TA Instruments).

## 3. Results and Discussion

### 3.1. Characterization of GNPs

**Figure 1:**
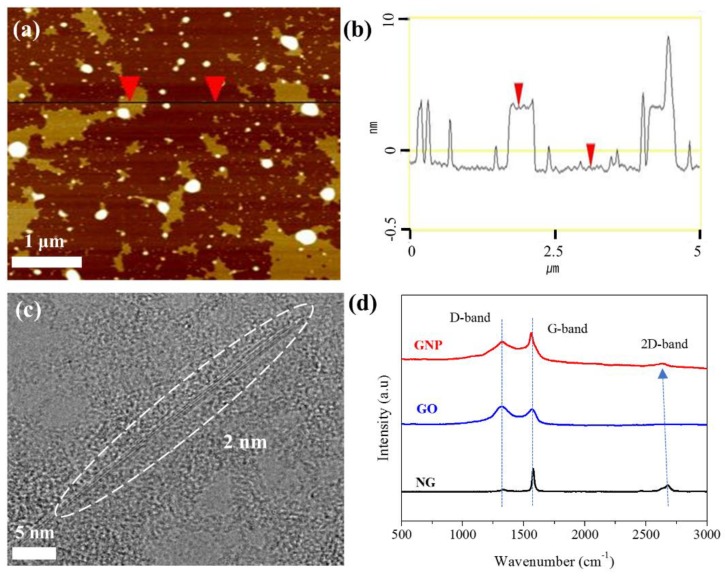
Characterization of the GNPs stabilized by CTAB: (**a**) AFM image of GNP; (**b**) Line profile of the AFM image; (**c**) TEM image of GNP; (**d**) Raman spectra of natural graphite (NG), graphite oxide (GO), and GNP after the removal of CTAB.

To produce GNPs with few structural defects, NG was exfoliated by liquid-phase exfoliation using CTAB. The optimum exfoliation yield of NG to GNP was found to be near the critical micelle concentration of CTAB after sonication for 24h as described shown in Figure S1 [35,36]. Finally, 0.35 mg/mL of the GNP/DI water solution was obtained after sonication for 24 h. Figure 1a–c show the AFM and TEM images of the GNPs that were obtained after sonication for 24 h. The thickness of GNP was 4 nm in AFM (Figure 1b) while it was 2 nm in TEM image. The difference is attributable to the CTAB adsorbed on GNP surface in AFM. The AFM and TEM images revealed that the average thickness and average size of GNPs are 2–3 nm and 0.15 μm^2^, respectively, as per the size-distribution analysis of a low-magnification TEM image given in Figure S2. These results revealed that GNPs with a few-layer structure are successfully obtained by the sonication-assisted liquid-phase exfoliation of NG and that their restacking is also effectively prevented in the presence of CTAB as a cationic surfactant.

As Raman spectroscopy has been widely employed to estimate the unique properties of graphite, including the amounts of structural defects and graphite layers; the intensity ratio of the D-band (~1350 cm^−1^) to the G-band (1580 cm^−1^) (*I*_D_/*I*_G_), as well as the 2D-band (2649–2677 cm^−1^), were estimated to investigate the quality and number of layers in the prepared GNPs [37,38,39,40]. Figure 1d shows the Raman spectra of NG, GO, and GNPs and the results are summarized in Table 2. Before Raman analysis, CTAB used for the exfoliation from NG to GNPs was removed using KOH without the addition of any chemical or polymer. Subsequently, the GNPs were washed and dried for removing the residual CTAB, KOH, and DI water to obtain pure GNPs. Due to the defects introduced in GNPs during the sonication, the *I*_D_/*I*_G_ of the GNPs was slightly larger than that of NG. However, *I*_D_/*I*_G_ of GNPs was much smaller than that of GO prepared by chemical oxidation of NG. In addition, FTIR analysis revealed that the exfoliation of NG by sonication in aqueous CTAB solutions does not lead to the changes in FTIR spectrum of the GNP as shown in Figure S3. These results suggested that although the graphitic structural damage of the *sp*^2^ domains is introduced during mechanical exfoliation by sonication, the process in aqueous CTAB solution results in less damage in GNPs compared with the chemical exfoliation process via oxidation. To further characterize the exfoliation of graphite, the shift in the 2D band was examined. The shift in the 2D band induced from a two-phonon double resonance process can be exploited for the prediction of the exfoliation degree [39,40]. The 2D band of NG was detected at 2677 cm^−1^, whereas that of GNP was detected at 2649 cm^−1^. This shift in the 2D band clearly corresponded to the exfoliation of NG, which reflected the decreased number of layers in multilayered NG. The 2D band position of GNP at 2649 cm^−1^ corresponded to 3–5 layers of graphite sheets. This value is almost consistent with the results obtained from TEM and AFM analyses even when GNPs were slightly restacked while removing CTAB. Hence, high-quality GNPs obtained by physical exfoliation are applied to produce the masterbatch and nanocomposites.

### 3.2. Preparation of GNP/Polyol Masterbatch

**Figure 2:**
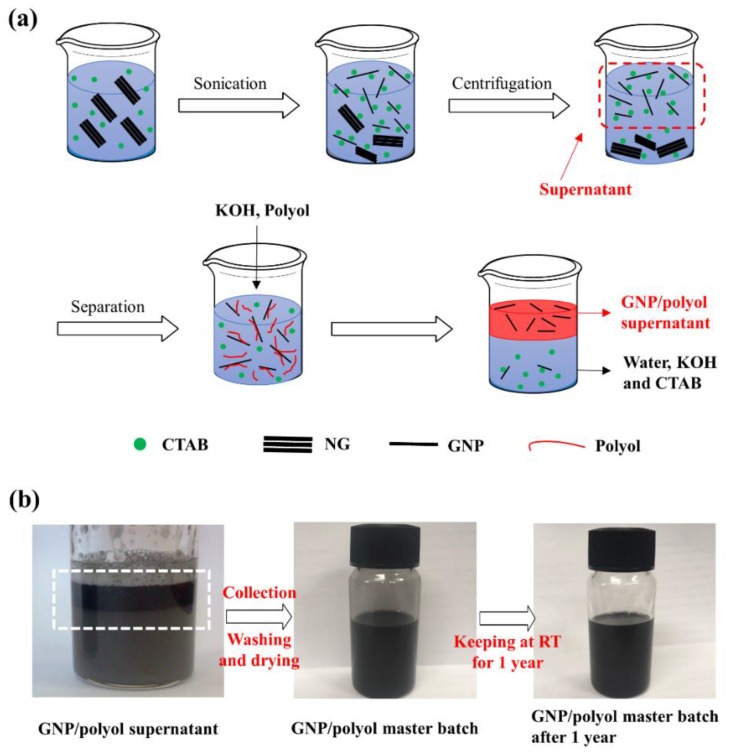
(**a**) Schematic of the separation of the GNP/polyol supernatant from an NG/GNP/CTAB/water solution, and (**b**) digital images of the GNP/polyol supernatant and storage stability of the GNP/polyol masterbatch after 1 year.

The obtained GNP/CTAB dispersion in DI water was successfully converted into the masterbatch by a simple process, as shown and described in Figure 2a and Section 2.3. The aqueous CTAB solution used for exfoliating NG was successfully replaced with polyol, which is a raw material used for PU synthesis without any chemical treatment. After this simple treatment, the C–N stretching (1217 cm^−1^) band, corresponding to the presence of CTAB, was not observed in the FTIR spectra of the masterbatch; moreover, bromine or nitrogen atoms were not observed in EDX data as shown in Figure S4, following the complete removal of CTAB in polyol. The concentration of GNPs in the masterbatch was determined as 6 wt% by TGA as shown in Figure S5. The prepared GPN/polyol masterbatch was confirmed to maintain excellent stability even after 1 year without any noticeable precipitation or aggregation of GNP (Figure 2b).

**Figure 3:**
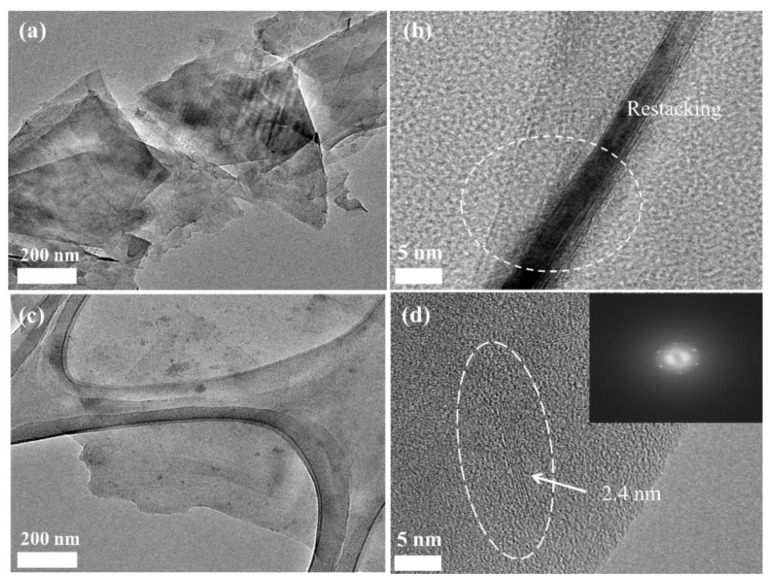
TEM images of the aggregated GNPs by KOH treatment (**a**,**b**), and TEM images of GNPs in the GNP/polyol masterbatch, (**c**,**d**).

Figure 3a,b shows the TEM images of the GNPs treated with KOH without polyol. As expected, after the washing of GNPs carried out without the addition of polyol, GNPs inevitably aggregated via van der Waals interactions among them because of CTAB removal. In comparison, washing was performed in the presence of polyol for the GNP/polyol masterbatch. The GNPs were apparently embedded in the polyol, and the thickness of GNPs was 2–4 nm (Figure 3c,d). This thickness range of GNPs was almost consistent with that of the initial GNPs, which were dispersed in the DI water solution with CTAB (Figure 1c). Furthermore, the thickness of GNPs in the GNP/polyol masterbatch kept for 1 year was also found to be 2–4 nm, which was similar to that of the as-prepared GNP/polyol masterbatch as shown in Figure S6. These results suggested that CTAB is successfully replaced by polyol, which prevented the aggregation of GNPs and facilitated the fine dispersion of GNPs for a long duration. Hence, the highly concentrated GNP/polyol masterbatch containing 6 wt% of high-quality GNPs with excellent storage stability is prepared by the replacement of aqueous CTAB with polyol, indicating that the GNP/polyol masterbatch can facilitate the preparation of GNP/PU nanocomposites with various concentrations of GNPs by simple dilution with additional polyol.

### 3.3. Lyotropic Liquid Crystalline (LLC) Behavior of GNPs in Polyol

**Figure 4:**
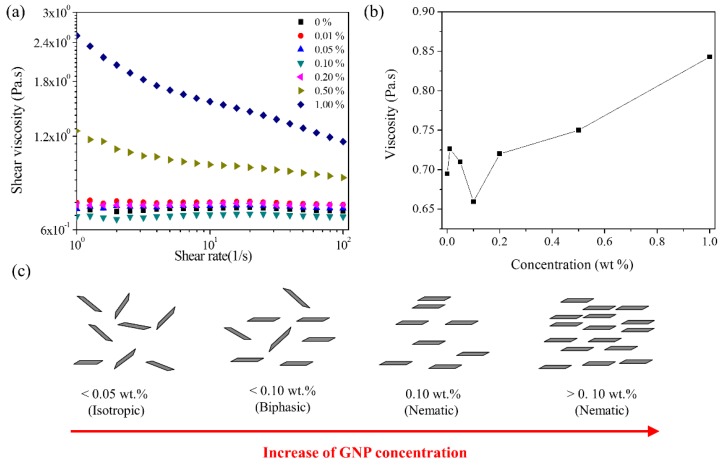
Rheological properties of the GNP dispersion in polyol diluted from the GNP/polyol masterbatch: (**a**) Shear viscosity versus the shear rate (1/s) of the GNP dispersion in polyol with various mass fractions of GNPs (from 0 to 1 wt%). (**b**) Shear viscosity with various GNP dispersions at 50 1/s; and (**c**) Schematic of GNPs in the polyols.

Figure 4a shows the shear viscosities versus shear rate curves of the GNP dispersion in polyol with different concentrations of GNPs prepared by the dilution of the GNP/polyol masterbatch with additional polyol. Figure 4b shows the shear viscosities for the different concentrations of the GNP dispersion in polyol at a shear rate of 50 1/s. The GNP dispersion exhibited typical LLC behavior depending on the GNP concentration due to the discotic nature (Figure 4c) [41,42,43,44]. The isotropic and biphasic dispersion at a low GNP concentration (<0.1 wt%) corresponded to randomly aligned GNPs. However, the nematic phase at 0.1 wt% of the GNP dispersion exhibiting the minimum viscosity corresponded to the parallel alignment of the GNPs in polyol [41,42]. The further increase in the viscosity at higher GNP concentrations (>0.1 wt%) corresponded to the dense parallel alignment of GNPs [44]. This LLC behavior of GNPs is not surprising. Several studies have reported that chemically modified GO exhibits LLC behavior in an aqueous dispersion [41,42,43,44]. However, notably, the GNP dispersion exhibiting LLC behavior in polyol can be directly used for manufacturing nanocomposites. Chemical reactions did not occur among GNPs and PU molecules due to the absence of chemically reactive groups on GNPs. The alignment of GNPs in polyol by LLC behavior possibly affected the morphology and mechanical properties of PU/GNP nanocomposites considerably.

### 3.4. PU/GNP Nanocomposites

#### 3.4.1. LLC Behavior of GNPs in PU Nanocomposites

**Figure 5:**
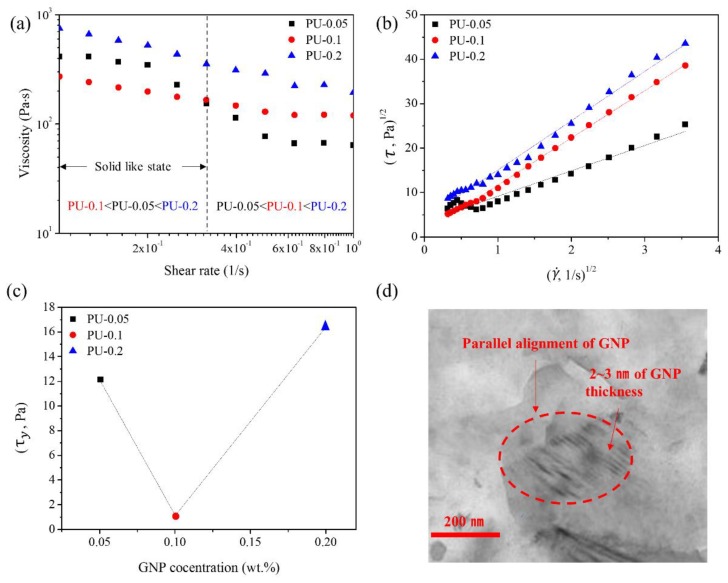
Characteristics of the GNP/PU nanocomposites. (**a**) Melt flow viscosities of GNP/PU nanocomposites at 180 °C. (**b**) Plots of $\tau^{1/2}$ versus ${\dot{\gamma }}^{1/2}$ to calculate the yield stress, $\tau_{y}$, of the GNP/PU nanocomposites. (**c**) Change of yield stress with GNP concentrations in PU nanocomposites. (**d**) GNP alignment of PU-0.1 observed by TEM.

By the simple dilution of the GNP/polyol masterbatch, the GNP dispersed polyol was directly used to prepare PU/GNP nanocomposites, and the alignment of the GNPs in the PU matrix was investigated by melt flow viscosity measurements. Figure 5a shows the melt flow curves of PU-0.05, PU-0.1, and PU-0. At relatively high shear rates (>0.5 1/s), the viscosity of nanocomposites increased with the GNP concentration. However, at lower shear rates (<0.5 1/s) all of the PU/GNP nanocomposites exhibited yield behavior in the nanocomposites [45,46]. In general, the ordering of GNPs along the shear direction needed to overcome yield stress [45]. The observed rheological properties in a low-shear-rate region was assumed to result from the yield behavior of the PU nanocomposites. Notably, at low shear rates, the viscosity of the nanocomposite increased in the order of PU-0.1 < PU-0.05 < PU-0.2, which was consistent with those of the GNP/polyol dispersions at the same GNP concentration (Figure 4). The yield stress (*τ*_y_) of the GNP/PU nanocomposites was estimated by the following Casson equation [47];

(1) $$\tau^{1/2}=\tau_{y}^{1/2}+{\left(\eta_{s}\;\cdot \;\dot{\gamma }\right)}^{1/2},$$

where *τ* and $\eta_{s}$ represent the shear stress and shear viscosity, respectively, at a shear rate ($\dot{\gamma }$). From the plot shown in Figure 5b, the yield stresses ($\tau_{y}$) of the GNP/PU nanocomposites were estimated. The yield stress was found to be in the order of PU-0.1 < PU-0.05 < PU-0.2, indicating that the alignment of the ordered structure of GNPs along the shear direction requires a small yield stress with parallelly aligned GNPs in the PU matrix (Figure 5c). The TEM image of PU-0.1 also clearly revealed the parallel alignment of GNPs in the PU matrix in the solid state (Figure 5d). Based on these results, the LLC dispersion of GNPs is also observed in the same concentration range in the polyol and PU matrix in Figure 4 and Figure 5. Kim et al. have reported that when the GO dispersion was applied to the formation of polymer nanocomposites, the GO concentration for LLC formation is possibly different due to the significant difference in polarities between the polymer and GO solution [48]. However, the GNPs used in this study were uniformly dispersed in polyol and in the PU matrix of the nanocomposites. GNPs were directly used without further treatment for manufacturing nanocomposites via bulk processing. The GNP concentration did not considerably differ with respect to the LLC behavior in both polyol and the PU matrix.

#### 3.4.2. Degree of Hydrogen Bonding and Microphase Separation of Nanocomposites

**Figure 6:**
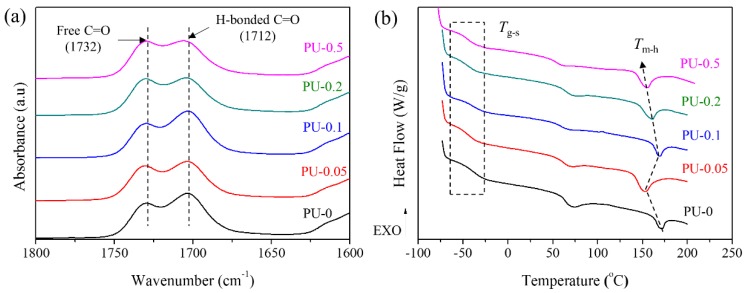
Characterization of the PU/GNP nanocomposites. (**a**) Fourier-transform infrared spectroscopy (FTIR) spectra at 1800–1600 cm^−1^. (**b**) Differential scanning calorimetry (DSC) thermograms during heating.

To verify the manner in which the LLC behavior of GNPs affected the structures of the PU matrix, FTIR spectra and DSC profiles were recorded. The FTIR spectra of the neat PU and GNP/PU nanocomposites were similar in the entire range of wavenumbers as chemical reactions did not occur between the PU matrix and GNPs as shown in Figure S7a. However, a clear difference was observed with respect to the intensities of C=O peaks at 1712 and 1732 cm^−1^, corresponding to hydrogen-bonded C=O and free C=O urethane linkages, respectively in Figure 6a. This result indicated that the GNP concentration considerably affects the conformation of the PU matrix, leading to different degrees of hydrogen bonding (R*) and degrees of microphase separation (DPS) of soft and hard segments of PUs. Hence, the R* and DPS of PU/GNP nanocomposites are estimated using the following equations [49];

A_1712_/A_1732_ = R* (2)

DPS = R*/(R* + 1) × 100%(3)

where A_1712_ and A_1732_ denote the absorbance of the peaks observed at 1712 and 1732 cm^−1^ in the FTIR spectra, and R* is the ratio of A_1712_ and A_1732_.

Table 3 summarizes the results of these analyses. The R* and DPS values slightly decreased with the increase in the GNP concentration in general because the randomly or partially ordered GNP dispersions not only disturbed the association among the hydrogen-bonding moieties but also introduced the phase mixing effect on the microphase-separated hard and soft segments of the PU matrix. However, PU-0.1 with parallel aligned GNPs exhibited R* (1.25) and DPS (55.5%) values similar to those of neat PU (PU-0, R* = 1.23, DSP = 55.1%). This result indicated that the parallel alignment of GNPs in PU-0.1 is the condition for GNP dispersion in the PU matrix with a small change in the microphase separation compared to neat PU. In addition, the thermal properties of nanocomposites were examined by DSC and given in Figure 6b and Table 3. Evident changes in glass transition temperatures of soft segments (*T*_g-s_) were not observed in the presence of GNPs. However, the melting points of hard segments (*T*_m-h_) clearly decreased but enthalpy change at *T*_m-h_ (∆*H*_m-h_) significantly increased for PU-0.05, PU-0.2, and PU-0.5, indicative of the disturbance to the microphase separation by the GNPs dispersed in the PU matrix. In addition, the increased crystallization temperature (*T*_c_) of hard segments was observed with the same samples as shown in Figure S7b because GNPs served as nucleating agents for the PU matrix [50,51]. These results suggested that the association of PU hard domains and their microphase separations are considerably affected by the introduction and alignment of GNPs in PU-0.05, PU-0.2, and PU-0.5. However, PU-0.1 exhibited similar *T*_m-h_ and *T*_c_ values to those of PU-0, indicating that the addition of 0.1 wt% GNP rarely affects the microphase separation of the PU matrix, which is consistent with the values of R* and DPS characterized by the FTIR spectra. 

#### 3.4.3. Mechanical Properties of GNP/PU Nanocomposites

**Figure 7:**
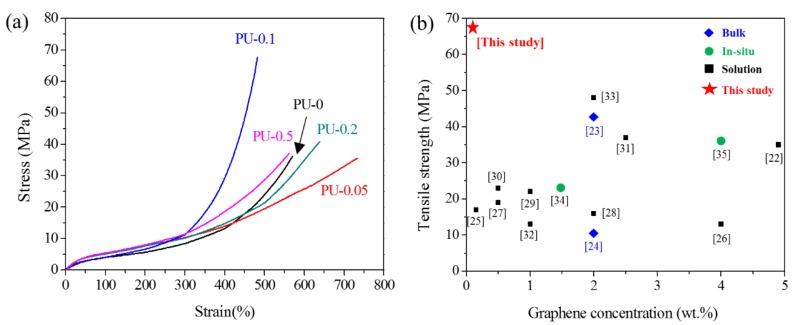
(**a**) Stress–strain curves of various GNP/PU nanocomposites. (**b**) The maximum tensile strength of graphite/PU nanocomposites using different preparation methods reported previously. The x-axis and numbers represent the concentration of graphene in the PU matrix, showing the maximum tensile strength, and number of references, respectively (Table S1).

Figure 7a shows the stress–strain curves, and Table 4 summarizes the tensile properties of PU/GNP nanocomposites. Considerable enhancement of the tensile strength and modulus were observed in the stress–strain curves upon the introduction of GNPs. The improved mechanical properties of nanocomposites are related to the high stiffness of GNPs and the successful stress transfer from the PU matrix to GNPs. In particular, PU-0.1 exhibited the maximum tensile strength (67.2 MPa) and Young’s modulus (10.60 MPa), with the fine dispersion of GNPs in the PU matrix. The large reinforcement of the tensile strength at 0.1 wt% of GNPs was considerable. To the best of our knowledge, the highest tensile strength of 67.2 MPa was noted among the PU/graphite nanocomposites reported previously as given in Figure 7b and Table S1. In particular, only 0.1 wt% of GNPs was required for the maximum reinforcement of tensile strength. Notably, the GNP/polyol masterbatch ensured the fine dispersion of GNPs in not only polyol but also the PU matrix even after polymerization without the assistance of any solvent [18,21,22,23,24,25,26,27,28,29,30,31] or the chemical treatment of graphite [18,19,20,21,22,23,24,25,26,27,28,29,30,31], leading to the excellent reinforcement effect in terms of the tensile strength of PU with only 0.1 wt% of GNPs.

**Figure 8:**
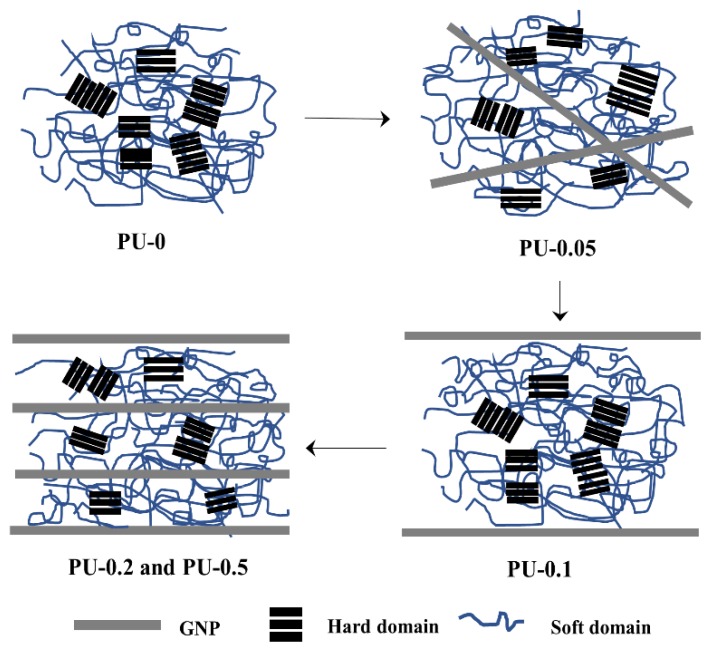
Schematic of the association among the hard domains of GNP/PU nanocomposites with differently aligned GNP according to GNP concentrations.

Furthermore, the remarkable improvement in the mechanical strength of PU-0.1 was related to the reinforcement effect of GNPs, with the preserved microphase separation of PU-0.1 being similar to that of neat PU. Compared to PU-0, PU-0.1 exhibited similar DPS and R* values in the FTIR spectra and *T*_m-h_ and *T*_c_ in DSC thermograms. However, those of PU-0.05, PU-0.2, and PU-0.5 exhibited large differences. The addition of 0.1 wt% GNPs that were aligned parallel in the PU matrix did not adversely affect the physical association among the hard and soft domains of PU, considerably improving the mechanical properties in combination with the reinforcing effect of GNPs as shown in Figure 8. However, with the random dispersion of the GNPs at a low concentration (PU-0.05) or alignment with the densely packed structure at high concentrations (PU-0.2 and PU-0.5), the physical association of the PU domains was apparently disturbed considerably by GNPs, with a large change in the conformation of the PU matrix, thereby decreasing R* and DPS, as well as changing the thermal properties in DSC. Specifically, *T*_m-h_ significantly decreased, which was strong evidence for the relatively lowered association of hard domains by the introduction of GNPs.

## 4. Conclusions

In this study a highly concentrated GNP/polyol masterbatch containing 6 wt% of high-quality GNPs was successfully manufactured by an eco-friendly method. The prepared masterbatch effectively prevented the aggregation of GNPs, which was easily applied for the production of PU/GNP nanocomposites by simple dilution. Interestingly, the GNPs exhibited LLC behavior in polyol, which were retained even at the GNP/PU nanocomposite. The alignment of GNPs in the nanocomposites considerably affected the conformation and microphase separation of the PU matrix, significantly affecting the mechanical properties of nanocomposites. Interestingly, the dispersion of GNPs at a specific concentration (PU-0.1) did not significantly affect the microphase separation, hydrogen bonding, or thermal properties of the microphase-separated PU compared to neat PU. Thus, the mechanical properties of PU-0.1 are remarkably improved via the effective reinforcing effect of GNPs in the nanocomposites.

## Figures and Tables

**Table 1:** Sample codes and compositions of the graphite nanoplate/polyurethane (GNP/PU) nanocomposites.

Sample Code	Composition (wt%)			
	MDI	Polyol	BD	GNP
PU-0	31.47	62.87	5.66	0
PU-0.05	31.45	62.84	5.66	0.05
PU-0.1	31.43	62.81	5.66	0.10
PU-0.2	31.40	62.74	5.65	0.20
PU-0.5	31.31	62.56	5.64	0.50

**Table 2:** *I*_D_/*I*_G_ ratios and location of the 2D band of NG, GO, and GNP.

Sample	Raman Characterization	
	*I*_D_/*I*_G_	2D Band (cm^−1^)
NG	0.1	2677
GO	1.2	NA
GNP	0.6	2649

**Table 3:** R*, degrees of microphase separation (DPS, %), and thermal properties for the PU/GNP nanocomposites of different GNP concentrations.

Sample Code	FTIR Spectroscopy		DSC			
	R*	DPS (%)	*T*_g-s_ (°C)	*T*_m-h_ (°C)	Δ*H*_m-h_ (J/g)	*T*_c_ (°C)
PU-0	1.23	55.1	−47	169	4.05	36
PU-0.05	1.08	51.9	−52	153	5.57	61
PU-0.1	1.25	55.5	−48	168	4.02	38
PU-0.2	1.01	50.2	−50	158	4.77	50
PU-0.5	1.03	50.7	−50	154	4.85	50

**Table 4:** Mechanical properties of GNP/PU nanocomposites.

Sample Code	Young’s Modulus (MPa)	Tensile Strength (MPa)	Elongation at Break (%)
U-0	4.2 ± 0.25	33.1 ± 1.49	570 ± 17.82
PU-0.05	7.5 ± 0.23	35.6 ± 1.23	740 ± 12.51
PU-0.1	10.6 ± 0.34	67.2 ± 1.21	470 ± 18.52
PU-0.2	7.3 ± 0.28	44.8 ± 1.42	640 ± 20.24
PU-0.5	8.7 ± 0.52	36.5 ± 0.82	560 ± 25.07

## Supplementary Materials

The following are available online at https://www.mdpi.com/2079-4991/9/3/389/s1, Figure S1: (a) Schematic process to prepare graphite nanoplate (GNP) from natural graphite (NG) by liquid phase exfoliation with CTAB, (b) UV-vis absorption spectrum of GNP dispersion stabilized by 0.1 wt% of CTAB, and (c) concentration (C) changes of GNP dispersions obtained from NG with CTAB of different concentration: (■) 0.05; (●) 0.075; (▲) 0.1; (▼) 0.5; and (◄) 1 wt%, Figure S2: (a) TEM images of GNPs dispersed in aqueous solution and (b) Size distribution of GNPs based on TEM images. The average size of GNPs was determined to be 0.15 μm^2^, Figure S3: FTIR spectra of NG and GNP collected from the process shown in Figure S1(a), Figure S4: (a) FTIR spectra of CTAB, polyol, GNP/polyol master batch and (b) the result of EDX analysis of GNP/polyol master batch, Figure S5: TGA curves of the CTAB (a), PTMEG-1000 (b), and GNP/polyol master batch, Figure S6: (a) and (b) are TEM images of GNP in GNP/polyol master batch which kept at room temperature for 1 year, Figure S7: Characteristics of PU/GNP nanocomposites; (a) FTIR spectra; (b) DSC thermograms obtained by cooling, Table S1: The tensile strengths and reinforcing efficiencies in the PU/GNP nanocomposites.
