# Supplementary material for: Large Improvement in the Mechanical Properties of Polyurethane Nanocomposites Based on a Highly Concentrated Graphite Nanoplate/Polyol Masterbatch
Source: Nanomaterials (Basel). 2019 Mar 7;9(3):389. doi: 10.3390/nano9030389 (PMC6474002; doi:10.3390/nano9030389)
Supplement: Supplementary file 1 [file nanomaterials-09-00389-s001.pdf]

nanomaterials

Large Improvement in the Mechanical Properties of Polyurethane Nanocomposites Based on a Highly Concentrated Graphite Nanoplate/Polyol Masterbatch

Sang-Hyub Lee†, Cho-Rong Oh† and Dai Soo Lee*

Division of Semiconductor and Chemical Engineering, Chonbuk National University, 567 Baekje-daero, Jeonju, 54896, Korea

Supplementary Materials

Table of Contents

1. **Exfoliation of GNP from NG by Sonication..S2**
2. **Characterization of GNP..S3**
3. **Characterization of GNP/polyol Masterbatch.....................................S4**
4. **Characterization of PU/GNP Nanocomposites..................................S5**

1. Exfoliation of GNP from NG by Sonication

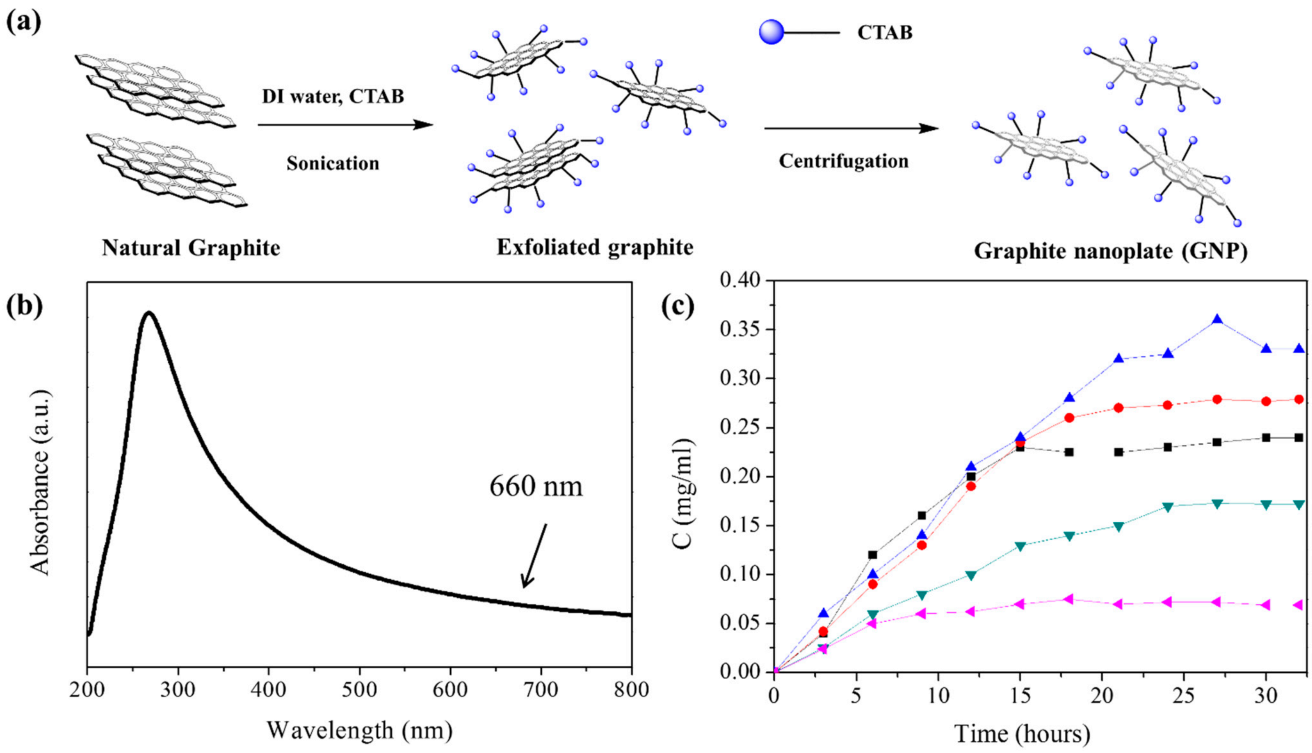

Figure S1. (a) Schematic process to prepare graphite nanoplate (GNP) from natural graphite (NG) by liquid phase exfoliation with CTAB, (b) UV-vis absorption spectrum of GNP dispersion stabilized by 0.1 wt% of CTAB, and (c) concentration (C) changes of GNP dispersions obtained from NG with CTAB of different concentration: (■) 0.05; (●) 0.075; (▲) 0.1; (▼) 0.5; and (◄) 1 wt%.

The concentration of GNP dispersion was obtained by UV-vis absorbance at 660 nm of the CTAB stabilized aqueous GNP dispersion employing Bear's law given in the following equation;

$$A = \varepsilon \times b \times C \qquad \text{(S1)}$$

where A is the absorbance at the specific wavelength (660 nm) and ε is an extinction coefficient of GNP dispersion in water, 1390 mL/mg [34,35], C is the concentration of GNP in dispersion, and b is the width of quartz cuvette used for the measurement. The concentration of GNP was estimated by equation S1 and given in Figure S1(c). It was found that the yield of GNP was greatly affected by sonication time and surfactant concentration. In Figure S1(c), the yield of GNP gradually increased with increasing the sonication time from 0 to 33 h. However, the yield tends to levelled-off at 24 h generally. It was worthwhile to note that the yield showed maximum at CTAB concentration of 0.1 wt%. The yield was largely decreased with increasing the concentration of CTAB beyond 0.1 wt%. The critical micelle concentration (CMC) value of CTAB was 0.125 wt% in CTAB/water solution [36]. This value is close to the concentration wt%of CTAB (0.1 wt%) showing the maximum yield for the GNP from NG by liquid phase exfoliation. It is speculated that CTAB formed micelles above CMC value in NG/water solutions instead of adsorption onto the GNP to stabilize in the aqueous systems. We could establish the optimum conditions for the concentration of CTAB and sonication time as 0.1 wt% and 24 h.

2. Characterization of GNP

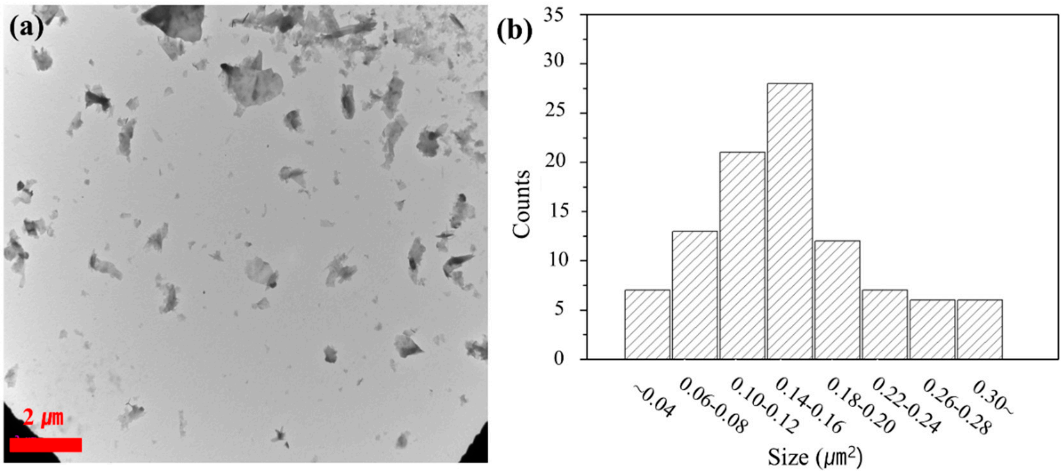

Figure S2. (**a**) TEM images of GNPs dispersed in aqueous solution and (**b**) size distribution of GNPs based on TEM images. The average size of GNPs was determined to be 0.15 μm².

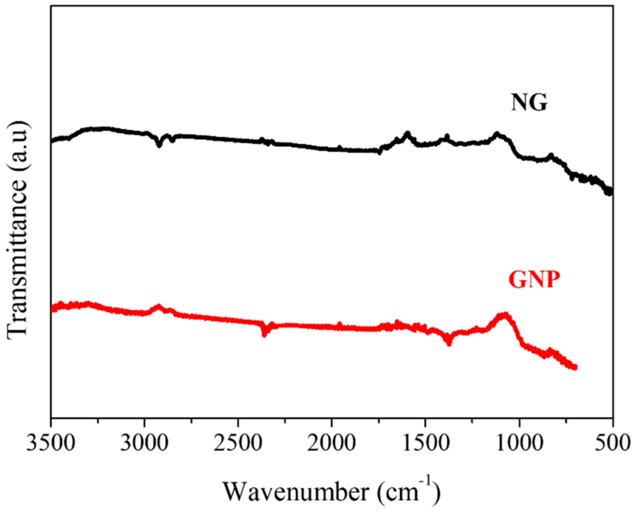

Figure S3. FTIR spectra of NG and GNP collected from the process shown in Figure S1(a).

3. Characterization of GNP/polyol Masterbatch

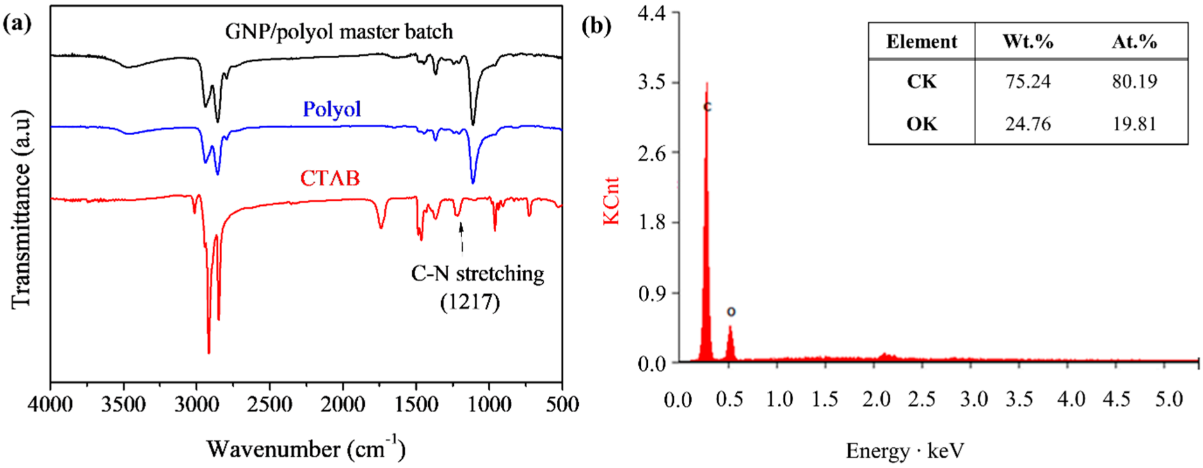

Figure S4. (**a**) FT-IR spectra of CTAB, polyol, GNP/polyol masterbatch and (**b**) the result of EDX analysis of GNP/polyol masterbatch.

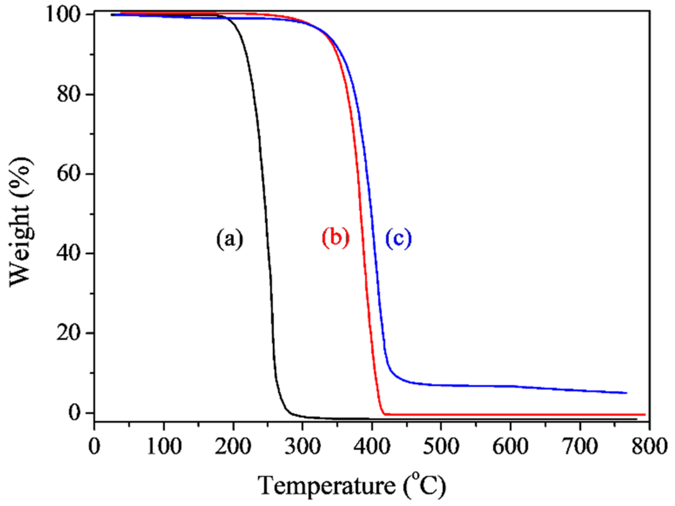

Figure S5. TGA curves of the CTAB (**a**), PTMEG-1000 (**b**), and GNP/polyol masterbatch.

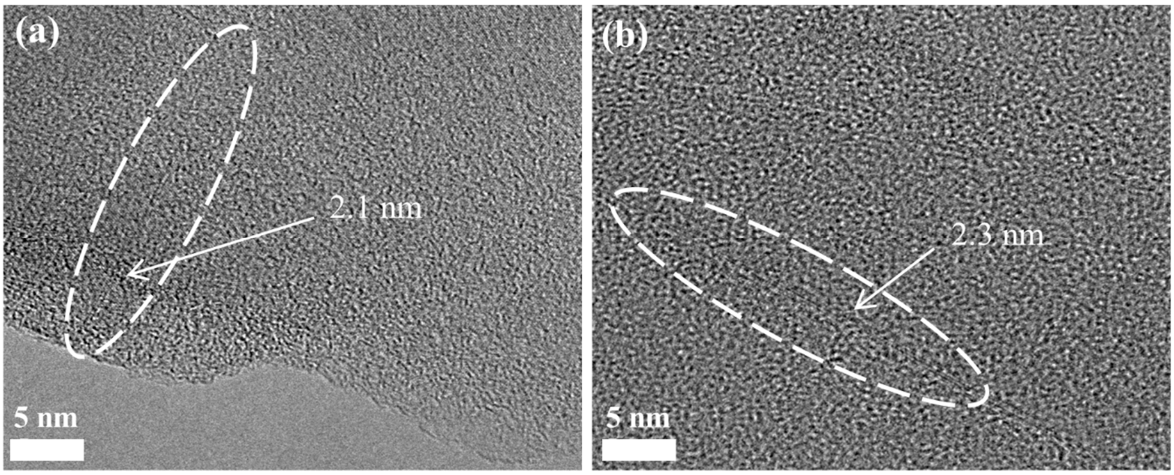

Figure S6. (**a**) and (**b**) are TEM images of GNP in GNP/polyol masterbatch which was kept at room temperature for 1 year.

4. Characterization of GNP/PU Nanocomposites

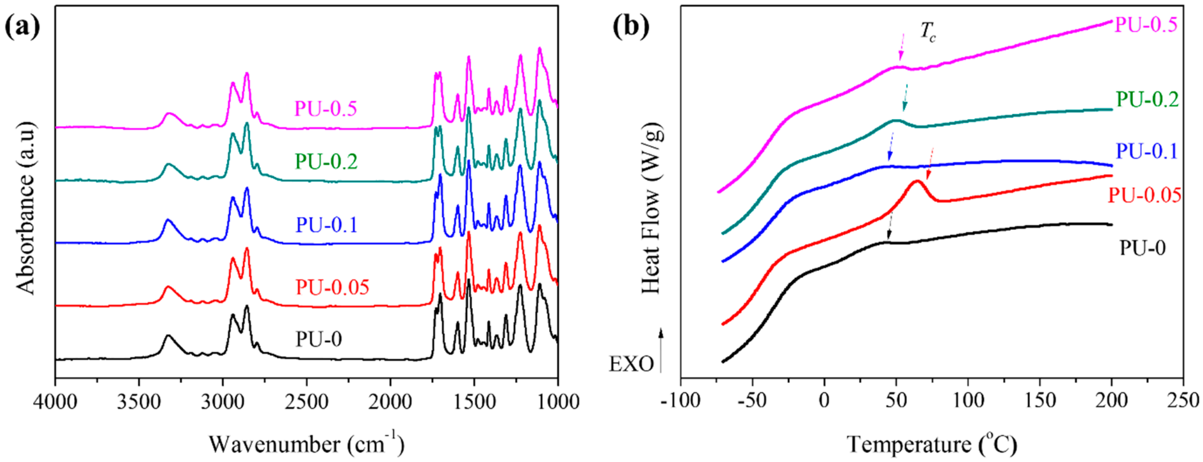

Figure 7. Characteristics of PU/GNP nanocomposites; (**a**) FTIR spectra; (**b**) DSC thermograms obtained by cooling.

Table S1. The tensile strengths and reinforcing efficiencies in the PU/GNP nanocomposites.

Type of graphene [a]	Optimum loading [b] (wt%)	Preparation method	Tensile strength (MPa)	Reinforcing efficiency (%)	Ref.
GNP	0.1	Bulk	67.21	102.8	This study
FGO	2	Bulk	42.68	17	[19]
TRGO	2	Bulk	10.6	70.75	[20]
FGO	0.15	Solution (DMF)	17	31	[21]
GO	4	Solution (DMF)	13	23.8	[22]
TRGO	0.5	Solution (MEK)	18.9	−5.5	[23]
GO	2	Solution (DMAc)	16.11	128.2	[24]
GO	5	Solution (DMF, THF)	35	40	[18]
RGO	1	Solution (DMF)	21.98	−24.26	[25]
FGO	0.5	Solution (DMF)	23	64.3	[26]
FGO	2.5	Solution (DMF)	37	105	[27]
FGO	1	Solution (DMF)	13.1	81.9	[28]
FGO	2	Solution (DMF)	48.1	3.59	[29]
GNP	1	In situ polymerization	19.5	8.93	[30]
F-GNP	1.5	In situ polymerization	23.4	30.7	[30]
RGO	4	In situ polymerization	36.3	236	[31]

a) GNP: graphene nanoplate, F-GNP: Functionalized graphene nanoplate, GO: Graphene oxide, FGO: Functionalized graphene oxide, TRGO: Thermally reduced graphene oxide, RGO: Reduced graphene oxide.

b) The optimum loading corresponds to the graphene loading where the tensile strength shows the maximum in the reports.
